# Frequency of HIV testing among gay and bisexual men in the UK: implications for HIV prevention

**DOI:** 10.1111/hiv.12373

**Published:** 2016-03-15

**Authors:** LM McDaid, A Aghaizu, J Frankis, J Riddell, A Nardone, D Mercey, AM Johnson, GJ Hart, P Flowers

**Affiliations:** ^1^ MRC/CSO Social and Public Health Sciences UnitUniversity of GlasgowGlasgowUK; ^2^ Public Health EnglandLondonUK; ^3^ Glasgow Caledonian UniversityGlasgowUK; ^4^ University College LondonLondonUK

**Keywords:** HIV prevention, HIV testing, men who have sex with men, sexual health, sexual risk behaviour

## Abstract

**Objectives:**

The aim of the study was to explore HIV testing frequency among UK men who have sex with men (MSM) in order to direct intervention development.

**Methods:**

Cross‐sectional surveys were completed by 2409 MSM in Edinburgh, Glasgow and London in 2011 and a Scotland‐wide online survey was carried out in 2012/13. The frequency of HIV testing in the last 2 years was measured.

**Results:**

Overall, 21.2% of respondents reported at least four HIV tests and 33.7% reported two or three tests in the last 2 years, so we estimate that 54.9% test annually. Men reporting at least four HIV tests were younger and less likely to be surveyed in London. They were more likely to report higher numbers of sexual and anal intercourse partners, but not “higher risk” unprotected anal intercourse (UAI) with at least two partners, casual partners and/or unknown/discordant status partners in the previous 12 months. Only 26.7% (238 of 893) of men reporting higher risk UAI reported at least four tests. Among all testers (*n* = 2009), 56.7% tested as part of a regular sexual health check and 35.5% tested following a risk event. Differences were observed between surveys, and those testing in response to a risk event were more likely to report higher risk UAI.

**Conclusions:**

Guidelines recommend that all MSM test annually and those at “higher risk” test more frequently, but our findings suggest neither recommendation is being met. Additional efforts are required to increase testing frequency and harness the opportunities provided by biomedical HIV prevention. Regional, demographic and behavioural differences and variations in the risk profiles of testers suggest that it is unlikely that a “one size fits all” approach to increasing the frequency of testing will be successful.

## Introduction

Men who have sex with men (MSM) are the group at highest risk of acquiring HIV in the UK, and an estimated one in five HIV‐positive MSM is undiagnosed 1. Mathematical modelling suggests that increasing the uptake of HIV testing and its frequency combined with antiretroviral treatment could reduce the incidence of HIV infection 2, 3, 4. Testing those at high risk every 3 months is cost saving when compared with annual testing 5. However, in the UK, HIV incidence is not decreasing 1, 6 and high proportions of newly diagnosed men have not previously tested 7, 8. Increases in the uptake of HIV testing in high‐income countries have been widely reported 9, 10, 11, 12, 13, 14, 15, but we have demonstrated a stabilization in recent HIV testing among MSM in Scotland, which suggests that the current opt‐out testing approach (whereby all patients are offered a test regardless of symptoms or risk factors) has reached its limit in maximizing routine uptake 16. Innovative methods of increasing the uptake of testing are required 4. Investigations of the psychosocial, sociocultural and technological aspects of testing will enable interventions to be developed which reduce barriers to testing, and promote frequent testing among high‐risk MSM 17.

Current British HIV Association/British Association of Sexual Health and HIV and Centers for Disease Control and Prevention guidelines recommend at least annual HIV testing for MSM 18, 19, with more frequent testing (up to every 3 months) recommended for individuals at risk of HIV acquisition 18. Critically, there is no consensus regarding the definition of those “at risk” or how to measure HIV testing frequency.

Frequent testing is likely to be central to the success of biomedical prevention approaches such as pre‐exposure prophylaxis (PrEP) and treatment as prevention (TasP) 20, 21, 22, 23, and in order to meet their challenges it is important that we are able to measure testing frequency. Self‐testing kits for HIV have been approved by the US Food and Drug Administration 4, and in the UK regulations outlawing their sale were repealed in April 2014 and commercial products became available in April 2015. However, it remains to be seen whether self‐testing kits can increase HIV testing and antiretroviral therapy uptake, particularly among those who would benefit most from it. While an international systematic review of research regarding self‐testing has demonstrated its acceptability 24, there are problems concerning the generalizability of its findings; many of the contributing studies focus upon developing countries and MSM are under‐represented. Moreover, most studies relate to an earlier era in the HIV epidemic when PrEP and TasP were unavailable 25, 26. In this paper we explore the frequency of, and reasons for, HIV testing among three community‐based samples of MSM in the UK, presenting estimates of annual and more frequent testing for the first time. We examine the factors associated with frequency of testing to assess the implications for future HIV prevention efforts.

## Methods

Data from three cross‐sectional surveys were examined.

- The Medical Research Council (MRC) Gay Men's Sexual Health Survey in Glasgow and Edinburgh collected anonymous, self‐complete questionnaires and oral fluid specimens (using OraSure^®^ Oral Specimen Collection Devices; OraSure Technologies, Inc., Bethlehem, PA, USA) in 17 gay commercial venues in May 2011, using a form of time and location sampling 9. Overall, 1515 men participated [65% response rate (RR)] and 1218 provided oral fluid samples (52% RR).
- The Glasgow Caledonian University (GCU) Social Media, MSM & Sexual Health Survey was a Scotland‐wide online survey carried out from November 2012 to February 2013. Pop‐up message blasts and/or banner adverts invited men using gay‐specific social media websites (Gaydar, Recon and Squirt), smartphone apps (Grindr and Gaydar) and Facebook to participate. In total, 1326 men completed useable questionnaires (given the nature of online surveys it is not possible to calculate a response rate).
- The University College London/Public Health England (UCL/PHE) Gay Men's Survey in London was conducted between March and June 2011 in 31 gay social venues including bars, clubs and saunas. Overall, 1216 men participated (RR 62%) and 1005 provided an oral fluid specimen using OraSure^®^ (RR 51%).

Ethical approval was granted by the University of Glasgow College of Social Sciences Ethics Committee (Glasgow/Edinburgh survey), the Health and Community Science subcommittee, the School of Health and Life Sciences Ethics Committee, Glasgow Caledonian University (Scotland‐wide online survey), and the University College London Hospital Research Ethics Committee (London survey).

The surveys included comparable data on demographics (age, area of residence and education), HIV/sexually transmitted infection (STI) testing, and sexual behaviour in the previous 12 months [numbers of sexual, anal intercourse (AI) and unprotected anal intercourse (UAI) partners]. Number of sexual and AI partner variables were dichotomized (< 10 *vs* ≥ 10 partners). A measure of UAI with higher risk for HIV infection was derived to include men who reported UAI with at least two partners, casual partners and/or unknown/discordant partners in the previous 12 months (compared with men reporting UAI with fewer than two partners, regular partners or known/concordant partners only).

Participants were asked how often they tested for HIV as described below, from which we calculated a measure of testing frequency indicative of annual and more frequent HIV testing. In the Glasgow/Edinburgh and London surveys, the number of HIV tests in the last 2 years was categorized into fewer than two, two or three and at least four tests. Testing regularity was sought in the Scotland‐wide online survey, so men who indicated that they tested “every 3/6 months” were categorized as having at least four tests, and those indicating testing “every year/every few years” were categorized as having had two or three tests; those remaining were categorized as having fewer than two tests. The latter category includes men who had never tested (who were a small proportion of the overall sample).

Participants were also asked the reasons for their last HIV test. Testing as a result of an episode of unprotected sex, condom error/accident, and/or sexual partner change was attributed to a perceived risk event, while testing as a result of a regular/routine sexual health check or offer from a health professional was coded as part of a regular sexual health check. Other reasons (e.g. visa requirements, blood donation, in vitro fertilization/sperm donation, other medical treatment, or life insurance applications) were coded as “other”. Categories were complicated by multiple responses, but our measure is hierarchical in that testing as a result of a risk event was prioritized over routine testing, and the latter was prioritized over “other”.

We excluded: HIV‐positive men (*n* = 134); men who did not provide an OraSure^®^ test in the Glasgow/Edinburgh/London surveys and did not therefore have confirmed HIV status (*n* = 297); men with missing data on the HIV status question in the Scotland‐wide online survey (*n* = 185); men who did not identify as gay or bisexual, because of the small number of responses (*n* = 59); and men who did not answer the question on when was their last HIV test and/or how often they tested for HIV (*n* = 599). Data were analysed using IBM spss 19.0 for Windows (IBM United Kingdom Limited, Portsmouth Hampshire, UK). *χ* ^2^ tests were used for bivariate comparisons. Multinomial logistic regression was conducted to compare the frequency for testing categories and binary logistic regression was used to compare men tested as part of a regular sexual health check and those tested in response to a perceived risk event. We adjusted for factors significant at the bivariate level (*P* < 0.05) and for demographic and behavioural differences between the surveys.

## Results

### Sample characteristics

The total sample was 2409. The mean age of participants was 34.2 years [range 18–83 years; standard deviation (SD) 11.1 years]. Most identified as gay (as opposed to bisexual) and reported education after age 16 years (Table 1). Just 9.8% had never had an HIV test, while 57.2% had tested in the previous 12 months; 14.3% reporting having an STI in the previous 12 months. Most reported sexual contact in the previous 12 months and 37.8% reported higher risk UAI in the previous 12 months (i.e. UAI with at least two partners, casual partners and/or unknown/discordant partners).

**Table 1:** Sample characteristics and differences between the Glasgow/Edinburgh, Scotland‐wide online, and London surveys (*n* = 2409)

	Glasgow/Edinburgh (*n* = 951)		Scotland‐wide online (*n* = 675)		London (*n* = 783)		Total (*n* = 2409)		*P*‐value
	*n*	%	*n*	%	*n*	%	*n*	%	
Age (years)									
≤ 25	291	30.8	135	20.0	152	19.6	578	24.1	< 0.001
26–35	334	35.3	186	27.6	329	42.3	849	35.4	
36–45	196	20.7	149	22.1	205	26.4	550	23.0	
≥ 46	124	13.1	204	30.3	91	11.7	419	17.5	
Sexual orientation									
Gay	878	92.3	578	85.6	749	95.7	2205	91.5	< 0.001
Bisexual	73	7.7	97	14.4	34	4.3	204	8.5	
Employment status									
Other	194	20.4	73	10.8	104	13.4	371	15.4	< 0.001
Employed	757	79.6	600	89.2	675	86.6	2032	84.6	
Educated post 16 years									
No	128	14.4	106	15.7	51	6.6	285	12.2	< 0.001
Yes	759	85.6	569	84.3	726	93.4	2054	87.8	
HIV testing									
Tested in previous 12 months	565	59.4	300	49.3	474	60.8	1339	57.2	< 0.001
Tested 1–5 years ago	281	29.5	126	20.7	200	25.6	607	25.9	
Tested over 5 years ago	58	6.1	47	7.7	59	7.6	164	7.0	
Never tested	47	4.9	136	22.3	47	6.0	230	9.8	
STI in previous 12 months									
No	815	86.2	578	86.4	661	84.4	2054	85.7	0.48
Yes	131	13.8	91	13.6	122	15.6	344	14.3	
Had sexual contact in previous 12 months									
No	26	2.8	16	2.4	21	2.9	63	2.7	0.83
Yes	917	97.2	652	97.6	702	97.1	2271	97.3	
Number of sexual partners in previous 12 months									
< 10	697	73.9	406	60.8	472	65.3	1575	67.5	< 0.001
≥ 10	246	26.1	262	39.2	251	34.7	759	32.5	
Had AI in previous 12 months									
No	146	15.5	83	12.4	60	8.4	289	12.4	< 0.001
Yes	795	84.5	585	87.6	656	91.6	2036	87.6	
Number of AI partners in previous 12 months									
< 10	830	88.2	530	79.3	533	74.4	1893	81.4	< 0.001
≥ 10	111	11.8	138	20.7	183	25.6	432	18.6	
Had UAI in previous 12 months									
No	454	48.2	338	51.3	333	44.9	1125	48.1	0.06
Yes	487	51.8	321	48.7	408	55.1	1216	51.9	
Higher risk UAI in previous 12 months^a^									
No	603	63.7	366	54.3	499	67.3	1468	62.2	< 0.001
Yes	343	36.3	308	45.7	242	32.7	893	37.8	

AI, anal intercourse; STI, sexually transmitted infection; UAI, unprotected anal intercourse.

a UAI with two or more partners, UAI with casual partners, and/or UAI with unknown/discordant partners.

Significant differences in the age patterning of the three surveys were apparent, with the highest proportion of young men (aged < 25 years) in the Glasgow/Edinburgh survey and the highest proportion of older men (aged ≥ 46 years) in the Scotland‐wide online survey. Participants in the latter survey had the highest mean age (37.8 years; SD 12.6 years), followed by the London survey (33.4 years; SD 9.5 years), while the participants in Glasgow/Edinburgh had the lowest mean age (32.3 years; SD 10.7 years) f(2) = 52.64; *P* < 0.001. The Scotland‐wide online survey also included a higher proportion of bisexual men. Men in the Glasgow/Edinburgh survey were least likely to report that they were employed, although the proportion with no education after age 16 years was highest in the Scotland‐wide online survey. Men in the Scotland‐wide online survey were more likely to have never tested for HIV, while testing in the previous 12 months was highest in the London survey. Significantly lower proportions of men in the Glasgow/Edinburgh survey reported ≥ 10 sexual or AI partners in the previous 12 months, but the difference in the proportions reporting any UAI between the surveys was borderline significant. The London survey sample was most likely to report having had an STI in the previous 12 months, but the proportion reporting higher risk UAI was highest in the Scotland‐wide online sample.

### Frequency of HIV testing

Overall, 510 men (21.2%) reported having at least four HIV tests, 812 (33.7%) reported having two or three tests, and 1087 (45.1%) reported having zero or one test in the last 2 years, and we estimate that 54.9% (*n* = 1322) had at least one test per year. Table 2 compares the characteristics of men reporting fewer than two tests, two or three tests, and at least four tests in the last 2 years. The Scotland‐wide online sample included the highest proportions of men reporting two or three tests and at least four tests in the last 2 years. Significantly higher proportions of older men (aged ≥ 36 years) reported zero or one test in the last 2 years, while reporting at least four tests was most common among younger men (aged ≤ 25 years). There was less age variation in the proportions reporting two or three tests. Men reporting higher risk behaviours (STI, ≥ 10 sexual partners, ≥ 10 AI partners and/or higher risk UAI in the previous 12 months) were consistently more likely to report at least four tests in the last 2 years than men who did not report these behaviours. Other demographic and behavioural variables showed no significant relationship with testing frequency.

**Table 2:** Factors associated with frequency of HIV testing in previous 2 years (zero or one test, two or three tests or at least four tests) among gay and bisexual men in the UK (*n* = 2409)

	Frequency of testing			*P*‐value
	0–1 test in last 2 years (*n* = 1087)	2–3 tests in last 2 years (*n* = 812)	≥ 4 tests in last 2 years (*n* = 510)	
	*n* (%)	*n* (%)	*n* (%)	
Survey				
Glasgow/Edinburgh	444 (46.7)	300 (31.5)	207 (21.8)	< 0.001
Scotland‐wide online	253 (37.5)	243 (36.0)	179 (26.5)	
London	390 (49.8)	269 (34.4)	124 (15.8)	
Age (years)				
≤ 25	223 (38.6)	196 (33.9)	159 (27.5)	< 0.001
26–35	361 (42.5)	292 (34.4)	196 (23.1)	
36–45	290 (52.7)	180 (32.7)	80 (14.5)	
≥ 46	207 (49.4)	141 (33.7)	71 (16.9)	
Sexual orientation				
Gay	1004 (45.5)	737 (33.4)	464 (21.0)	0.41
Bisexual	83 (40.7)	75 (36.8)	46 (22.5)	
Employment status				
Other	171 (46.1)	126 (34.0)	74 (19.9)	0.83
Employed	915 (45.0)	683 (33.6)	434 (21.4)	
Educated post 16 years				
No	130 (45.6)	95 (33.3)	60 (21.1)	0.97
Yes	927 (45.1)	700 (34.1)	427 (20.8)	
STI in previous 12 months				
No	952 (46.3)	699 (34.0)	403 (19.6)	< 0.001
Yes	129 (37.5)	111 (32.3)	104 (30.2)	
Number of sexual partners in previous 12 months				
< 10	806 (51.2)	506 (32.1)	263 (16.7)	< 0.001
≥ 10	250 (32.9)	278 (36.6)	231 (30.4)	
Number of AI partners in previous 12 months				
< 10	918 (48.5)	642 (33.9)	333 (17.6)	< 0.001
≥ 10	134 (31.0)	138 (31.9)	160 (37.0)	
Higher risk UAI in previous 12 months^a^				
No	704 (48.0)	504 (34.3)	260 (17.7)	< 0.001
Yes	360 (40.3)	295 (33.0)	238 (26.7)	

AI, anal intercourse; STI, sexually transmitted infection; UAI, unprotected anal intercourse.

a UAI with two or more partners, UAI with casual partners, and/or UAI with unknown/discordant partners.

Multinomial logistic regression analysis was used to compare the three testing groups (Table 3). First we considered the comparison between men reporting zero or one test and those reporting two or three tests in the last 2 years. The two groups differed significantly on survey location and age. Those reporting two or three tests were more likely to come from the Scotland‐wide online survey and they were less likely to be aged > 36 years than ≤ 25 years. Those reporting two or three tests were also more likely to have had ≥ 10 sexual partners in the previous 12 months. Next we compared men reporting zero or one test and at least four tests in the last 2 years. Those reporting at least four tests were more likely to come from the Scotland‐wide online survey and less likely to come from the London survey or to be aged > 36 years. There were additional differences with respect to sexual behaviour: men reporting at least four tests were more likely to report having ≥ 10 sexual partners, ≥ 10 AI partners and an STI in the previous 12 months. There was no significant association with higher risk UAI. Finally, when comparing men reporting two or three tests and at least four tests, those reporting at least four tests were less likely to come from the London survey or be aged > 36 years and more likely to report ≥ 10 AI partners in the previous 12 months. Those reporting at least four tests were more likely to have higher risk UAI in the previous 12 months but this was of borderline statistical significance (*P* = 0.05).

**Table 3:** Multinomial logistic regression comparing men reporting zero or one test in the last 2 years, two or three tests in the last 2 years and at least four tests in the last 2 years (*n* = 2409)

	2–3 tests *vs* 0–1 test in last 2 years			≥ 4 tests *vs* 0–1 test in last 2 years			≥ 4 tests *vs* 2–3 tests in last 2 years		
	AOR	95% CI	*P*‐value	AOR	95% CI	*P*‐value	AOR	95% CI	*P*‐value
Survey									
Glasgow/Edinburgh (reference)	1			1			1		
Scotland‐wide online	1.45	1.13–1.85	< 0.01	1.65	1.25–2.20	< 0.01	1.14	0.86–1.52	0.36
London	0.94	0.74–1.19	0.60	0.61	0.45–0.82	< 0.01	0.65	0.47–0.89	0.01
Age (years)									
≤ 25 (reference)	1			1			1		
26–35	0.97	0.74–1.26	0.82	0.79	0.59–1.07	0.13	0.82	0.61–1.11	0.20
36–45	0.68	0.51–0.90	0.01	0.35	0.25–0.50	< 0.001	0.52	0.36–0.76	< 0.01
≥ 46	0.70	0.51–0.96	0.03	0.39	0.27–0.58	< 0.001	0.56	0.38–0.83	< 0.01
Sexual orientation									
Gay (reference)	1			1			1		
Bisexual	1.07	0.75–1.53	0.70	1.07	0.70–1.64	0.74	1.00	0.66–1.53	0.99
Employment status									
Other (reference)	1			1			1		
Employed	1.00	0.79–1.31	0.98	1.16	0.82–1.62	0.40	1.16	0.82–1.64	0.41
Educated post 16 years									
No (reference)	1			1			1		
Yes	0.99	0.74–1.34	0.96	0.91	0.64–1.30	0.60	0.92	0.63–1.32	0.64
STI in previous 12 months									
No (reference)	1			1			1		
Yes	1.11	0.82–1.48	0.50	1.49	1.08–2.06	0.02	1.35	0.97–1.87	0.07
Number of sexual partners in previous 12 months									
< 10 (reference)	1			1			1		
≥ 10	1.89	1.43–2.49	< 0.001	1.86	1.32–2.61	< 0.001	0.99	0.71–1.38	0.94
Number of AI partners in previous 12 months									
< 10 (reference)	1			1			1		
≥ 10	0.95	0.67–1.36	0.80	2.10	1.41–3.13	< 0.001	2.20	1.50–3.24	< 0.001
Higher risk UAI in previous 12 months^a^									
No (reference)	1			1			1		
Yes	0.90	0.73–1.12	0.36	1.16	0.91–1.49	0.23	1.29	1.00–1.66	0.05

AOR, adjusted odds ratio; CI, confidence interval; AI, anal intercourse; STI, sexually transmitted infection; UAI, unprotected anal intercourse.

a UAI with two or more partners, UAI with casual partners, and/or UAI with unknown/discordant partners.

### Reasons for HIV testing

We explored the reasons that men who had ever tested provided for having their most recent HIV test (*n* = 2009). Overall, 1140 (56.7%) tested as part of a regular sexual health check, 714 (35.5%) reported testing in response to a perceived risk event, and 155 (7.7%) tested for other reasons. In comparing the characteristics of the three groups (Table 4), we found that the Glasgow/Edinburgh survey had the highest proportion tested as part of a sexual health check, the London survey had the highest proportion tested after a perceived risk event, and the Scotland‐wide online sample had the highest proportions tested for other reasons (this survey also included the most alternative testing options). Reasons for testing varied with age, with the highest proportion tested after a perceived risk event in the youngest age category (≤ 25 years). Men reporting higher risk UAI in the previous 12 months were more likely to report testing in response to a perceived risk event, although little variation in other sexual risk behaviours was observed. As would be expected, the proportion reporting testing as part of a regular check‐up increased with the frequency of HIV testing.

**Table 4:** Factors associated with reasons for last HIV test among gay and bisexual men who have had an HIV test in the UK: *n*, row %, unadjusted and multivariate logistic regression comparing men tested as part of a regular test or sexual health check‐up and men tested in response to a risk event (*n* = 2009)

	Reason for last HIV test			Comparison of men tested as part of a regular test/sexual health check‐up and men tested in response to a risk event					
	Testing as part of regular testing/sexual health check (*n* = 1140)	Testing in response to risk event (*n* = 714)	Other reasons^a^ (*n* = 155)	Unadjusted logistic regression			Multivariate logistic regression		
	*n* (%)	*n* (%)	*n* (%)	OR	95% CI	*P*‐value	AOR	95% CI	*P*‐value
Survey									
Glasgow/Edinburgh	547 (63.2)	285 (32.9)	34 (3.9)	1			1		
Scotland‐wide online	258 (55.0)	132 (28.1)	79 (16.8)	0.98	0.76–1.27	0.89	1.03	0.79–1.34	0.84
London	335 (49.7)	297 (44.1)	42 (6.2)	1.71	1.39–2.11	< 0.001	1.74	1.40–2.17	< 0.001
Age (years)									
≤ 25	252 (53.5)	188 (39.9)	31 (6.6)	1			1		
26–35	430 (58.8)	254 (34.7)	47 (6.4)	0.76	0.60–0.97	0.03	0.72	0.56–0.93	0.01
36–45	255 (54.3)	175 (37.2)	40 (8.5)	0.89	0.68–1.16	0.37	0.83	0.62–1.10	0.19
≥ 46	199 (61.2)	90 (27.7)	36 (11.1)	0.63	0.46–0.85	< 0.001	0.63	0.46–0.88	0.01
Sexual orientation									
Gay	1047 (56.8)	662 (35.9)	135 (7.3)	1					
Bisexual	93 (56.4)	52 (31.5)	20 (12.1)	0.91	0.64–1.29	0.60	–	–	–
Employment status									
Other	194 (60.6)	109 (34.1)	17 (5.3)	1					
Employed	943 (56.0)	604 (35.9)	137 (8.1)	1.13	0.87–1.45	0.36	–	–	–
Educated post 16 years									
No	134 (58.3)	75 (32.6)	21 (9.1)	1					
Yes	965 (56.0)	627 (36.4)	130 (7.5)	1.16	0.86–1.56	0.34	–	–	–
STI in previous 12 months									
No	972 (57.0)	598 (35.1)	134 (7.9)	1					
Yes	164 (55.0)	114 (38.3)	20 (6.7)	1.12	0.87–1.45	0.37	–	–	–
Number of sexual partners in previous 12 months									
< 10	752 (57.2)	461 (35.1)	102 (7.8)	1					
≥ 10	359 (56.8)	225 (35.6)	48 (7.6)	1.04	0.85–1.27	0.71	–	–	–
Number of AI partners in previous 12 months									
< 10	902 (57.1)	553 (35.0)	125 (7.9)	1					
≥ 10	202 (56.1)	131 (36.4)	27 (7.5)	1.07	0.84–1.37	0.57	–	–	–
Higher risk UAI in previous 12 months^b^									
No	743 (60.5)	392 (31.9)	94 (7.6)	1			1		
Yes	376 (51.0)	301 (40.8)	60 (8.1)	1.56	1.29–1.90	< 0.001	1.67	1.37–2.05	< 0.001
Frequency of HIV testing									
0–1 test in last 2 years	446 (51.2)	344 (39.5)	81 (9.3)	1			1		
2–3 tests in last 2 years	420 (59.4)	241 (34.1)	46 (6.5)	0.74	0.60–0.91	< 0.01	0.71	0.57–0.89	< 0.001
≥ 4 tests in last 2 years	274 (63.6)	129 (29.9)	28 (6.5)	0.60	0.47–0.77	< 0.001	0.58	0.46–0.76	< 0.001

OR, odds ratio; AOR, adjusted odds ratio; CI, confidence interval; AI, anal intercourse; STI, sexually transmitted infection; UAI, unprotected anal intercourse.

a “other” category is excluded from the logistic regression analysis because of the small number of cases in each category.

b UAI with two or more partners, UAI with casual partners, and/or UAI with unknown/discordant partners.

Binary logistic regression was used to compare men tested as part of a regular sexual health check and those tested in response to a perceived risk event (Table 4). The odds of having had an HIV test because of a perceived risk event remained significantly higher among the London survey sample compared with Glasgow/Edinburgh, and among men reporting higher risk sexual behaviour than among men not reporting this behaviour. The adjusted odds were significantly lower among men aged ≥ 46 years and men aged 26–35 years, than among those aged ≤ 25 years, and among those reporting more frequent HIV testing in the previous 2 years. All variables remained significant in the multivariate model.

## Discussion

This is the first study to explore the frequency of HIV testing amongst MSM in the UK. Half reported at least two HIV tests in the last 2 years, suggestive of annual testing, the minimum recommended in current UK guidelines 18. However, fewer than one in five reported having four or more tests in the last 2 years, which suggests that 6‐monthly testing is less common. The guidelines recommend that all men test annually and those at “higher risk” test up to every 3 months, but our findings suggest neither recommendation is being met. Among the HIV testers, more than half reported that their most recent test was part of a regular sexual health check and over one‐third tested in response to a perceived risk event. Regional, demographic and behavioural differences are worthy of attention and each will be considered in turn.

Regional differences in HIV testing behaviour are not new and we are among those who have previously reported on such, particularly between the large urban centres of the UK 27, 28, 29. When compared with the Glasgow/Edinburgh survey, we found evidence of higher testing frequency in the Scotland‐wide online survey and lower frequency in the London survey. This was the case despite higher rates of recent testing in the Glasgow/Edinburgh and London samples, demonstrating the limitations of the recency measure and the different survey methods and sample characteristics. However, testing as a result of a perceived risk event was higher in the London survey. While between‐survey differences should be treated with caution, they suggest the need to consider regional differences in the roll‐out, uptake and potential impact of HIV testing interventions. For example, the promotion of frequent, regular testing was a particular focus of Scottish HIV prevention efforts (particularly on‐scene) 30, which could account for the higher levels of this behaviour. However, it is also possible that the variations reflect the demographic and behavioural differences evident between the samples.

Our results suggest that the frequency of HIV testing decreases with age and that there are differential patterns of risk among age groups. When comparing infrequent (zero or one test in the last 2 years) and annual testers (two or three tests in the last 2 years), there was a significant association with the number of sexual, but not AI, partners in the previous 12 months. When comparing annual and frequent testers (at least four tests in the last 2 years), the reverse pattern was observed in that there was a significant association with the number of AI partners but not the total number of sexual partners. Both number of sexual partners and number of AI partners were significant when comparing infrequent and frequent testers. Having an STI in the previous 12 months was only significantly different between infrequent and frequent testers. Although somewhat complicated and inconsistent, this does suggest a patterning by sexual risk behaviour similar to findings from elsewhere, albeit using different measures of frequency (studies in the USA and Australia have measured inter‐test intervals, and repeat and return testing) 31, 32, 33. However, higher risk UAI was not associated with testing frequency. Indeed, only one‐quarter of men reporting higher risk UAI also reported the frequent testing recommended (up to every 3 months) for those at high risk of HIV infection. This difference between guidelines and actual practice has also been reported in Australia, where only 34% were found to meet the comprehensive STI and HIV testing recommendations for sexually active gay and bisexual men 13. In another Australian study, 6‐monthly re‐testing rates were only 15% among higher risk MSM 33.

A strong association between frequent and regular testing has been reported elsewhere 31, and the lack of association between higher risk UAI and testing frequency could indicate that episodes of higher risk UAI are less frequent events, albeit reported by over one‐third of our sample. Furthermore, higher risk UAI was associated with testing after a perceived risk event (when compared with testing as part of a regular sexual health check), which suggests that men reporting higher risk UAI could be aware of the HIV‐related risk inherent in their behaviour and are testing accordingly. HIV prevention requires men to incorporate increasingly complex understandings of transmission risks and sero‐adaptive behaviours into their sexual lives 34, 35, 36. The extent to which men are able to do so and the level of sexual health literacy required to fulfil this task are largely unknown and worthy of further research. Furthermore, given that current guidelines suggest that individuals at risk of HIV test as frequently as every 3 months (as well as after a risk event) 18, and that men newly diagnosed with HIV are known to have been less frequent testers 1, 31, 37, 38, there is a clear need to promote frequent testing as a distinct and routinized behaviour through behaviour change interventions and to address barriers to frequent testing accordingly.

Most men who attend for a sexual health screen will have an HIV test 39. An audit of sexual health clinics in England found that almost all MSM reported one or more HIV tests in a 12‐month period 40, which does suggest that clinic attenders are meeting the minimum annual testing recommendations 18. Yet in our varied community samples, this recommendation was consistently not being met and it is likely that not all men are attending clinics for regular sexual health screening. Our data suggest subtle differences in the risk profiles of regular *vs* “risk event” testers, who are at potentially greatest risk for HIV infection. Accessing services remains a key opportunity for intervention and frequent testing for HIV and STIs should be promoted to those men only testing after a risk event. Annual testing should be conducted at a population level with MSM to reduce undiagnosed HIV infection and regular sexual health screening should be offered to all men at risk of STI/HIV transmission.

Caution should be adopted in generalizing our findings beyond the respective survey populations or to the wider population of MSM in the UK. All three surveys provide cross‐sectional data and causality cannot be inferred, and while the reliance on self‐report data could be subject to bias, this has been minimized by their anonymous, self‐complete nature; the surveys also have comparability and consistency over time. While the Glasgow/Edinburgh and London surveys included a biological measure of HIV status, the Scotland‐wide online survey could not and is reliant on self‐reported HIV status, which is therefore likely to include undiagnosed HIV‐positive participants. Possible underreporting of this should be noted, particularly as one‐quarter of the HIV‐positive men in the Glasgow/Edinburgh survey population are known to be undiagnosed 41. Minor differences in the wording of questions could have affected interpretations and the figures presented could be over‐ or under‐estimates of actual HIV testing rates. For the testing frequency measure, we assumed that two tests in the last 2 years was indicative of an annual test, but this may not be the case (i.e. both tests could have been in the last year). Further work is required to refine the measure of testing frequency. Similarly, the between‐survey differences, while of interest, should not be overemphasized in case they are in some part reflective of subtle variations in the mode of questioning or data collection methodologies, particularly between online and venue‐based samples 42. However, combining samples from different locations allows us to present a broader picture of men's experiences and HIV testing behaviours. As social and sexual mixing patterns change, and the use of online networking sites and other social media as a means of identifying and meeting sexual partners increases 43, 44, behavioural research will need to incorporate such multiple recruitment strategies.

HIV testing is a core component of current HIV prevention, but despite substantial increases in HIV testing in recent years, our results suggest that MSM in the UK do not test frequently enough. Evidence‐based behaviour change interventions are needed to increase the frequency of testing among those at risk for HIV infection. Such interventions will prove essential to facilitating the effectiveness of PrEP (PrEP requires individuals to have accurate knowledge of their HIV status) 45. Innovative means of increasing uptake are being tested 46, 47, 48, 49, 50, 51, but it is not yet clear if any of these approaches will increase testing frequency in the medium to longer term or in the subpopulations that matter. It is also unknown whether the availability of self‐testing, or even self‐sampling, kits will increase the frequency of testing, particularly in those at higher risk. The regional, demographic and behavioural differences, and the subtle variations in the risk profiles of testers described here make it unlikely that a “one size fits all” approach to increasing the frequency of testing will be successful. Our analysis suggests that targeted and tailored behaviour change interventions may well offer purchase to this complex problem. Moreover, additional efforts to reduce the known barriers to HIV testing remain important 52, 53, particularly if we are to optimize the potential of biomedical interventions.

## Author contributions

LM/PF devised the paper and LM wrote the first draft. JR undertook data analysis and contributed to drafts of the manuscript. All authors contributed to interpretation of the data, contributed revisions, and approved the final version of the manuscript.
